# Survival of dental implants and occurrence of mucosal overgrowth in patients with head and neck cancer treated with/without radiotherapy and mucosal graft—two-year follow-up

**DOI:** 10.1007/s00784-023-05479-0

**Published:** 2024-01-25

**Authors:** Viivi Mattila, Tommy Wilkman, Nina-Li Avellán, Karri Mesimäki, Jussi Furuholm, Hellevi Ruokonen, Karita Nylund

**Affiliations:** Department of Oral and Maxillofacial Diseases, HUS Helsinki University Hospital and University of Helsinki, Helsinki, Finland

**Keywords:** Dental implant, Head and neck cancer, Radiation therapy, Chemoradiotherapy, Oral squamous cell carcinoma

## Abstract

**Objectives:**

The primary aim of the present study was to compare head and neck cancer treatment modality surgery and surgery with radiotherapy or chemoradiotherapy alone for dental implant (DI) survival. The second aim was to evaluate the prevalence of mucosal overgrowth around DI after treatment with or without mucosal grafts.

**Materials and methods:**

An observational retrospective study consisted of 59 patients with malignant head and neck tumors that received DI between 2015 and 2019. Treatment modalities together with information on oral rehabilitation with DI, prevalence of mucosal overgrowth, and precursor lesions were gathered from the hospital records. Radiation doses were determined using a sum of three-dimensional dose distributions.

**Results:**

Overall DI survival rate was 88%, in irritated jaw 89%, and in nonirradiated jaw 88% in this observational period (*p* = 0.415, mean follow-up was 2 years 10 months, range 9–82 months). Mucosal overgrowth was found in 42 of 196 implants (21%), of which 36 cases (86%) were associated in grafted areas (*p* < 0.001). Oral lichen planus/lichenoid reaction was diagnosed in 14 of all 59 (24%) oral cancer patients.

**Conclusion:**

Implant survival was not significantly influenced by radiation therapy in this observational period. In grafted bone, implant survival was significantly inferior than in native bone. Mucosal overgrowth around implants was more common in mucosal grafted areas versus nongrafted.

**Clinical relevance:**

This study demonstrates the impact of grafted bone to dental implant survival rate and mucosal overgrowth.

## Introduction

Treatment of oral cancer is based on surgical therapy with or without radiotherapy or chemoradiotherapy [1]. Treatment options depend on several factors, such as the type and stage of the tumor and the patient’s general health. Surgical resection is the most well-established mode of initial definitive treatment for a majority of oral cancer patients, and in general, early-stage tumors are managed by a single-modality surgical treatment. Reconstructive surgery, mainly using microvascular free flaps, is often required to restore functional or esthetic loss of tissue.

Dental implant treatment is the preferred method of replacing missing teeth today. The survival rate of implant treatment varies from 90 to 97% during 5 to 10 years of follow-up in patients who are otherwise healthy and without oral cancer [2–4]. However, oral carcinoma patients may have specific features for prosthetic rehabilitation/reconstruction after ablative surgery and/or radiotherapy/chemoradiotherapy, as soft and hard tissue defects. These anatomical alterations after surgical treatment of cancer affect oral function, chewing, swallowing, speech, and esthetic appearance. In addition, radiotherapy and chemoradiotherapy may cause side effects such as trismus, bone healing problems, mucositis, xerostomia, taste disturbances, and further infections like caries, candida, and periodontal infections. The placement of DI-assisted rehabilitation prostheses in patients with oral cancer has shown a high degree of satisfaction and more effective oral rehabilitation. Patients’ quality of life has improved significantly [5]. Dental implant treatment among oral cancer patients is challenging, and the timing of implant placement after irradiation can affect implant survival.

The survival of dental implants placed in irradiated jaw bone in oral cancer treatment is controversial. Ettl and coworkers (2016) followed up with 39 head and neck cancer patients with 234 implants for 2 years. Overall, implant survival rate after 2 years was 92.3%. However, smoking, bone grafts, and radiation dose > 60 Gy were significant contributors to implant failure [6]. According to a systematic review by Kende et al. [7], the presence of radiotherapy does not play a significant role in the survival rate of DI in oral cavity cancer patients.

The aim of this retrospective study was to determine the survival rate of DI placed in 2015–2019 after head and neck cancer treatment in the Department of Oral and Maxillofacial Diseases Head and Neck Center, Helsinki University Hospital, Helsinki, Finland, and to compare cancer treatment modality surgery and surgery with radiotherapy or chemoradiotherapy alone for implant survival. The second aim was to evaluate the prevalence of mucosal overgrowth around DI after treatment with or without mucosal grafts and to register the prevalence of oral cancer precursor lesions lichen planus or lichenoid lesions among these patients.

## Materials and methods

This is a retrospective and descriptive study of malignant head and neck tumor patients treated with DI in 2015–2019 in a tertiary medical care unit, Department of Oral and Maxillofacial Diseases Head and Neck Center, Helsinki University Hospital, Helsinki, Finland. All patients who received DI between 2015 and 2019 after oral cancer treatment were included.

The patients were identified by searching the data from patient records of Helsinki University Hospital for the procedure codes EBB10 (implant placement) and EBB11 (next implant placement) and excluding other than head and neck cancer patients. Demographic data (age, sex, smoking status, systemic diseases, number of medications), cancer type, site, ICD-10 code, and treatment modalities together with information on oral rehabilitation with DI (implant system, site, timing of surgery, prosthetics), prevalence of mucosal overgrowth, and precursor lesions lichen planus or lichenoid lesions were gathered from the hospital records. Radiation doses (from sequential radiotherapy) were determined using a sum of three-dimensional dose distributions.

The oral health of patients before oral cancer treatment was evaluated using the modified total dental index (mTDI) calculated from the panoramic radiograph analyses of 32 teeth. mTDI describes the inflammatory burden caused by caries, periodontitis, and periapical- and pericoronitis lesions, and it ranges from 0 to 10 [8].

The index is calculated as follows: 1–3 caries lesions, 1 point; 4–7 lesions or no teeth in mandible or maxilla, 2 points; 8 or more lesions or radix or no teeth, 3 points. Alveolar bone loss is recorded as bone loss in the cervical third of a root, 1 point; in the middle third, 2 points; and in the apical third, 3 points. Periapical lesions are calculated as follows: one periapical lesion or vertical bone pocket or both, 1 point; two periapical lesions, 2 points; three or more periapical lesions, 3 points. Pericoronitis of partially erupted teeth is scored as follows: no pericoronitis, 0 point; or pericoronitis present, 1 point [8]. The patients were divided into groups on their mTDI score: low mTDI (0–2), medium mTDI (3–4), and high mTDI (≥ 5). The low mTDI group has a low number of dental infection, while the high mTDI group has a high number of dental infection.

The study was approved by the Helsinki and Uusimaa Hospital research committee (license number § 6 HUS/126/2021) and registered in the hospital database.

### Statistical analysis

We used the SPSS statistical software package for data analysis. For categorical variables, we evaluated differences in association between the study groups with Pearson’s chi-square test. Throughout the study, we considered *p*-values below 0.05 to be statistically significant.

## Results

### Patient demographics

The entire data consisted of 59 patients (cancer in oral cavity *N* = 50, other head and neck cancer *N* = 9). Of the 59 subjects, 32 were females (54%) and 27 were males (46%) with a mean age of 68 years (range 44–92). In addition, 36% (*N* = 21) of head and neck cancer patients had a history of tobacco smoking, 13 patients (13%) were smokers, and 25 patients were (42%) nonsmokers. Forty-six out of 59 (78%) patients had at least one systemic disease such as diabetes (*N* = 10), cardiovascular disease (*N* = 14), or asthma (*N* = 6). The majority of patients (*N* = 32) had mTDI three to four out of ten, representing a moderate oral infection burden, while 22 patients had low mTDI (0–2). However, five patients had a high oral infection burden with mTDI ≥ 5.

See Table 1 for patient demographics.

**Table 1:** Basic characteristics of the patients

Parameter	Oral cavity cancer *N* = 50 (100%)	Other head and neck cancer *N* = 9 (100%)	Total cancer *N* = 59 (100%)
Sex			
Male	20 (40%)	7 (78%)	27 (46%)
Female	30 (60%)	2 (22%)	32 (54%)
Smoking			
Yes	10 (20%)	3 (30%)	13 (22%)
No	23 (47%)	2 (20%)	25 (42%)
Ex-smoker	16 (33%)	5 (50%)	21 (36%)
Diabetes	11 (22%)	0 (0%)	11 (19%)
Cardiovascular diseases	10 (20%)	4 (44%)	14 (24%)
Other systemic diseases	23 (46%)	4 (44%)	27 (46%)
No systemic diseases	6 (12%)	7 (78%)	13 (22%)
Other cancers	9 (18%)	4 (44%)	13 (22%)
Number of medications			
Under 5	25 (50%)	7 (78%)	32 (54%)
5–10	16 (32%)	1 (11%)	17 (29%)
Over 10	9 (18%)	1 (11%)	10 (17%)

### Histopathology

The most common histopathologic tumor type identified was oral squamous cell carcinoma (OSCC) found in 54 out of 59 patients (92%), with 7 patients (12%) experiencing tumor recurrence. Other tumor types were epithelial-myoepithelial carcinoma (EMC) (*N* = 1, 2%), adenoid cystic carcinoma (*N* = 3, 5%), and clear cell adenocarcinoma (*N* = 1, 1%).

Oral lichen planus/lichenoid lesions were diagnosed in 14 subjects (24%), mostly in females (*N* = 13/14; 93%). Oral verrucous lesions were diagnosed in two subjects (3%), verrucous hyperplasia in one, and a proliferative verrucous leukoplakia (PLV) in one patient.

Seven patients had multifocal OSCC, and in six out of seven cases (86%), lichen planus/lichenoid lesions were found as a precursor lesion.

### Characteristics and treatment modalities of primary cancer

From 59 head and neck cancer patients, 50 cancers were detected in the oral cavity: 26 from gingiva (ICD-10 C03.1 *N* = 18, C03.0 *N* = 8), nine in the tongue (C02.11 *N* = 7, C02.2 *N* = 2) followed by floor of the mouth (C04.1 *N* = 1, C04.8 *N* = 1, C04.0 *N* = 5), palate (C05.8 *N* = 2, C05.0 *N* = 2), cheek (C06.0 *N* = 3), and base of tongue (C01 *N* = 2). The rest were found in the sublingual gland (C08.11), laryngopharynx (C13.8), palatine tonsils (C09.9, C06.8, C09.8), larynx (C32.8), and parotid gland (C07.7), whereas one was nonspecific (C77.0). Thirty out of 59 patients (51%) were treated by ablative surgery and radiotherapy, of which 12 were treated with chemoradiotherapy, 27 patients (46%) with ablative surgery alone, and two patients (3%) had definitive chemoradiotherapy between 1998 and 2019. Orofacial defects in 45 out of 59 patients (76%) were treated using microvascular reconstruction. Of these, eight had free microvascular bone reconstruction (two fibula, three deep circumflex iliac artery flap (DCIA), three modified minihybrid free flap composed of the anterolateral thigh, and a partial inner lamina iliac crest only (ALT/DCIA)), and five soft tissue free flaps (one radial forearm free flap (RFA), four anterolateral thigh free flap (ALT)) with nonvascularized bone transplant and 32 soft tissue free flaps (22 RFA, nine ALT, 1 medial sural flap surfacing remaining own jaw bone). Details of primary cancer treatment modalities can be found in Table 2.

**Table 2:** Characteristics and treatment modalities of the primary cancer

Location of primary	Oral cavity cancer (*n* = 50)	Other head and neck cancer (*n* = 9)	Total (*n* = 59)
Gingiva C03.1 C03.0	26 (52%) 18 (36%) 8 (16%)	-	26 (44%) 18 (31%) 8 (14%)
Tongue C02.11 C02.2	9 (18%) 7 (14%) 2 (4%)	-	8 (15%) 7 (12%) 2 (3%)
Cheek C06.0	3 (6%)	-	3 (5%)
Palate C05.8 C05.0	4 (8%) 2 (4%) 2 (4%)	-	4 (7%) 2 (3%) 2 (3%)
Floor of mouth C04.0 C04.1 C04.8	7 (14%) 5 (10%) 1 (2%) 1 (2%)	-	7 (12%) 5 (8%) 1 (2%) 1 (2%)
Base of tongue C01	-	2 (22%)	2 (4%)
Laryngopharynx C13.8	-	1 (11%)	1 (2%)
Palatine tonsils C09.9 C06.8 C09.8	-	3 (33%) 1 (11%) 1 (11%) 1 (11%)	3 (5%) 1 (2%) 1 (2%) 1 (2%)
Larynx C32.8	-	1 (11%)	1 (2%)
Nonspecific C77.0	-	1 (11%)	1 (2%)
Parotid gland C07.7	-	1 (11%)	1 (2%)
Sublingual gland C08.11	1 (2%)		1 (2%)
Treatment modality			
Surgery	27 (54%)	0 (0%)	27 (46%)
Surgery with radiotherapy	15 (30%)	3 (33%)	18 (31%)
Surgery with chemoradiotherapy	8 (16%)	4 (44%)	12 (20%)
Chemoradiotherapy as a single modality	0 (0%)	2 (22%)	2 (3%)
Reconstruction			
DCIA	3 (6%)	-	3 (5%)
Fibula flap	1 (2%)	-	1 (2%)
Modified hybrid DCIA/ALT	3 (6%)	-	3 (5%)
ALT with free nonvascularized bone	3 (6%)	-	3 (5%)
ALT	1 (2%)	-	1 (2%)
RFA	7 (14%)	-	7 (12%)
Suralis with radial forearm flap	1 (2%)	-	1 (2%)
RFA with free nonvascularized bone	1 (2%)	-	1 (2%)
Ulnaris and fibula	1 (2%)	-	1 (2%)

### Implant therapy and prosthetics

**Fig. 1:**
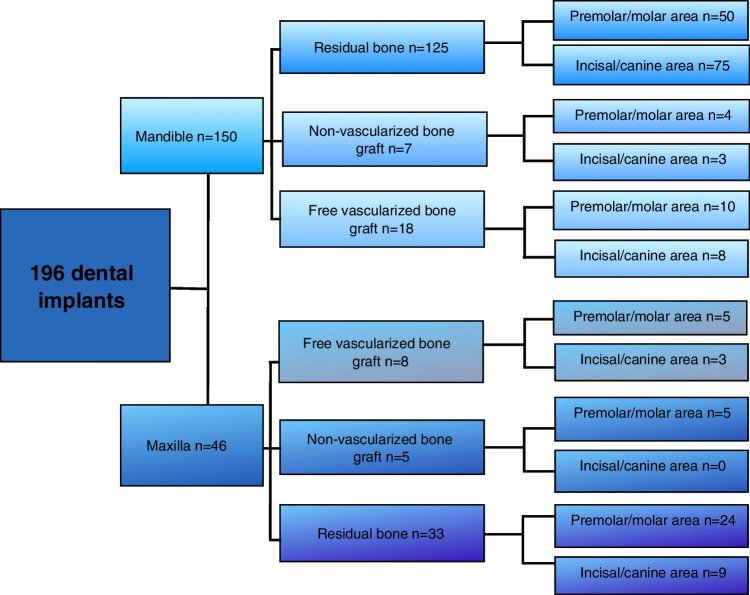
A flowchart showing 196 dental implants for oral rehabilitation

Between 2015 and 2019, a total of 196 DI were placed for oral rehabilitation. The number of DI placed in each patient ranged from 1 to 13 (mean 3.3). Out of 59 patients, 34 (58%) were partially edentulous and 25 (42%) were edentulous. Of these 196 implants, 150 were placed in the mandible (88 in the incisal/canine area and 62 in the premolar/molar area) and 46 DI were placed in the maxilla (12 in the incisal/canine area and 34 in the premolar/molar area). Seven implants were implanted in the fibula, ten implants to DCIA, and ten to ALT/DCIA. See Fig. 1 for details in the flowchart.

Thirty patients received postoperative radiotherapy. Twelve implants (in four patients) were placed prior to radiotherapy and 91 implants (in 26 patients) were placed after radiotherapy. The mean dose of radiotherapy was 61 Gy (range 50–70 Gy), and the mean time between the radiotherapy and implant surgery was 50 months (range of 9 to 240 months). Radiation therapy was given to three nonvascularized bone grafts, 12 vascularized bone grafts, and 72 to the patient’s own bone.

The mean follow-up period for the implant survival was 2 years and 10 months. Nine patients died during the observational period due to cancer metastasis, Covid-19 infections, pneumonia, and/or other infections.

A variety of DI brands such as Straumann, AstraTech, Xive, and Conelog were placed. Most DI were 4–5 mm in diameter and 9–12 mm in length. A total of 73 implant-supported prostheses were constructed for 56 patients. These included 15 fixed-bridge units, 32 overdentures (11 locator-retained and 21 bar-retained), two partial dentures, and 24 crowns. Seventy-one suprastructures were only implant-borne and two suprastructures were implant- and tooth-borne. Nine implants were not utilized, two were left as sleepers, five were present but not loaded, two DI could not be used for prosthetic rehabilitation due to implants mispositioned, and one patient died before a prosthesis could be provided. There was no evidence of fractures or failed prosthetic components.

### Dental implant survival rate

Overall DI survival rate was 88%, in irradiated bone was 89%, and in nonirradiated bone was 88% (*p* = 0.415, mean follow-up 2 years 10 months, range 9–82 months). Twelve implants had been placed before irradiation and three of those failed, with the survival rate being 75%. DI survival in irradiated grafted bone was 80% and in nonirradiated grafted bone was 82%.

A total of 38 implants were placed in grafted bone, 27 in vascularized bone, and 11 in nonvascularized bone. The survival rate for DI placed in grafted bone was 76% (free vascularized bone was 76% and nonvascularized was 75%) and the survival rate in nongrafted bone was 91% (*p* = 0.026).

The implant survival rate for smokers (*N* = 13) with 41 implants was 90% since four implants were lost during the observational period, implant survival rate for ex-smokers (*N* = 21) with 65 implants was 98% since one implant was lost, while nonsmokers (*N* = 25) with 80 implants lost 19 implants with a survival rate of 74% (*p* < 0.001).

Patients with low mTDI implant survival rate were 78% (11 implants were lost for 51 implants), while intermediate mTDI implant survival rate was 90% and high mTDI was 93% (*p* = 00.1).

Specifically, 24 implants (12%) were lost, seven before prosthodontic treatment, nine during the loading phase, and eight were lost due to the recurrence of oral cancer. Excluding the eight implants lost due to the recurrence of oral cancer, the DI survival rate increased to 91%. The reasons for DI failure excluding these recurrences were infection (*N* = 13) and mucosal or bone defects (*N* = 3). The mean time for implant loss was 20 months (range 6 to 67 months).

See Table 3 for survival rates.

**Table 3:** Survival rates

	Implants, *n*	Failures, *n*	Survival, %	*p*-value
Overall	196	24	87.78	
Irradiated bone	91	10	89.01	0.415
Nonirradiated bone	93	11	88.17	
Irradiated bone before implantation	12	3	75.00	
Irradiated grafted bone	15	3	80.00	0.450
Nonirradiated grafted bone	22	4	81.82	
Mandible	150	13	91.33	0.004
Maxilla	46	11	76.09	
Immediate	29	4	86.20	1.000
Delayed	167	20	88.02	
Native bone	158	15	90.50	0.026
Grafted bone	38	9	76.32	
1 stage	22	4	81.82	0.486
2 stage	174	20	88.50	
Smoker	41	4	90.24	< 0.001
Nonsmoker	80	19	74.32	
Ex-smoker	65	1	98.46	
Implants loaded	179	17	90.50	< 0.001
Implants not loaded	17	7	58.82	
Low mTDI (0–2)	51	11	78.43	< 0.001
Intermediate mTDI (3–4)	120	12	90.00	
High mTDI (> 5)	15	1	93.33	

### Mucosal overgrowth

Mucosal overgrowth around implants (total of 42 cases) was associated with 34 cases in the grafted area (bone and mucosal graft or mucosal graft), eight cases in native bone and mucosa, and 12 cases in the irradiated jaw. The incidence of mucosal overgrowth in the grafted area compared to the nongrafted area was statistically significant, *p* < 0.001. Most of the mucosal overgrowth cases (*N* = 21) were associated with RFA, eight cases with ALT, and seven cases with the fibula free flap.

## Discussion

The main finding in this retrospective, descriptive study of malignant head and neck tumor patients was that the overall dental implant survival rate was 88% (88% in nonirradiated and 89% in radiated jaw) in a mean of 2.8 years of observational period. Thus, no statistically significant difference was detected in DI survival rates between DI placed in irradiated or nonirradiated bone. Thirty-seven DI were placed in irradiated bone (radiation below 50 Gy) out of which four failed, with a survival rate of 89%. Twenty-two DI were placed in irradiated bone (radiation over 50 Gy), out of which seven failed, with a survival rate of 89%, so the radiation dose is not associated with lower DI survival rates in this retrospective study. Our finding is in line with the results of a systematic review by Kende et al. [7] where the survival rate was 82.47% and 89.37% in irradiated and nonirradiated jaws, respectively, with a mean follow-up period of 52.5 months. Also, Yerit et al. [9] and Schepers et al. [10] have shown that implant survival rate is high in irradiated bone after 18- to 24-month follow-up. According to Schiegnitz et al. [11], the survival rate of DI varied from 68 to 99% (mean 89%) in irradiated local bone, from 86 to 100% (mean 96%) in nonirradiated local bone, from 39 to 95% (mean 81%) in irradiated grafted bone, and from 75 to 100% (mean 92%) in nonirradiated grafted bone in oral cancer patients. The implant survival rate in our retrospective study in irradiated grafted bone was 80% and in nonirradiated grafted bone was 82%.

However, as stated, the survival of DI placed in irradiated jaws in oral cancer treatment is controversial. A study by Ettl and coworkers [5] showed that smoking, bone grafts, and radiation dose > 60 Gy were significant factors affecting implant failure when they followed up on 39 head and neck cancer patients with 234 implants for 2 years [6]. In the present retrospective study, smokers lost fewer DI than nonsmokers. The lower survival rate of implants in nonsmokers may be due to patient selection for implant treatment, short observational period, and the nonsmokers’ group containing patients with recurrent tumors. Smoking may influence more to success rate, which our study does not regard. According to the retrospective study by Shaw et al. [12] at the beginning of the follow-up period, there were similar primary implant losses in the irradiated and nonirradiated groups in head and neck cancer patients. Literature reveals that the implant survival rate in irradiated bone is lower than in nonirradiated bone in a long-term follow-up period [13, 14]. In the present study, the implant survival rate was lower in patients with low mTDI than in patients with intermediate mTDI or high mTDI. However, in the low mTDI group, 22 of 51 implants (43%) were placed in grafted bone, while in the intermediate mTDI group, 45 of 120 implants (38%) were placed in grafted bone. As shown in Table 3, implant survival was lower in the grafted bone vs. native bone (*p* = 0.026).

Radiotherapy has side effects for vascularization, osseointegration, and tissues’ ability to regenerate. As concluded by Kende et al. [7], the interval between the definitive therapy of oral cancer and the installation of DI may contribute to the failure of osseointegration; thus, delayed implant placement may have a better chance of survival.

As previously stated, reconstructive surgery, mainly using microvascular free flaps, is often required to restore functional or esthetic loss of tissue. Superficial defects of the mucosa can be left to secondary healing or reconstructed by local mucosal grafts. Larger defects require free tissue flaps. The fibula free flap, DCIA, scapula free flap, and RFA are the primary choices for mandibular osseous reconstructions. Also, ALT/DCIA can be used for reconstruction in the mandibular region [15]. Large defects of the upper gum alveolar region or hard palate can be reconstructed by using an osteocutaneous free flap from the fibula, iliac crest, or scapula, and in some cases with soft tissue free flap such as rectus abdominis or anterolateral thigh free flap. Radiotherapy/chemoradiotherapy is applied postoperatively in Finland in cases such as oropharyngeal cancer, especially HPV-related cancers as a single-modality treatment. Overall implant survival rate in grafted areas was poorer in our study compared to nongrafted areas (75% vs. 91%); thus, grafted areas proved to be a significant risk factor for implant loss. The lower survival of implants in the grafted area may be the result of differences in bone volume and soft tissue quality compared to the original residual bone and mucosa. When comparing grafted areas with nongrafted, grafted areas had more mucosal overgrowth. This may be the result of a loss of attached and keratinized mucosa and diminished oral hygiene of tumor patients.

In this study, five patients were unable to wear their prosthesis due to discomfort, six patients used a prosthesis despite problems with mastication, swallowing functions, and speech. Altered local anatomy, implant positioning, and loss of keratinized mucosa are this group’s implant challenges. Prosthetic rehabilitation is often difficult due to hyposalivation, mucosal atrophy, loss of lip competence, oronasal or oroantral communications, oral hygiene, poor swallowing function, tethered tongue, scar bands, poor access, limited mouth opening, and limited freeway space**.** The goal of placing implants in head and neck tumor patients is to enable sufficient oral rehabilitation and therefore try to increase their quality of life while not endangering their survival. Careful planning is an important part of successful implant dentistry. Patients’ cooperation, oral hygiene, and motor coordination of hands, bone, and soft tissue volume after tumor surgery impact the planning and survival of dental implants. Successful implant dentistry and implant prosthodontics are only possible with a team that works well together. Dental and maxillofacial surgeons, prosthodontists, periodontists, and dental technicians are essential partners in this cooperative effort. Even if the collaboration is good, however, some problems are unfortunately encountered after prosthetic rehabilitation.

Oral lichen planus/lichenoid lesions were diagnosed in 14 subjects (24%), mostly in females (*N* = 13/14; 93%). Alrashdan et al. [16] have reviewed the literature regarding the incidence of oral lichen planus which is most common in females between 30 and 60 years of age. Lichen planus/lichenoid lesions were detected as a precursor lesion in six out of seven multifocal OSCC. Mignogna et al. [17] reported that oral lichen planus patients have a risk for multiple and multifocal malignancies in the oral cavity.

The strength of the current study is that it was a single-center study and all patients were treated by the same regimen and guidelines. We also have the exact radiation doses.

One limitation of this study is that the survival of implants placed in 2015–2019 was followed only for a mean of 2.8 years from the hospital records. Another weakness is the fairly small number of patients and that the success of implants could not be recorded. The reason was that the whole oral status could not be used because all the clinical measurements had not been recorded systematically. As is known, the survival rate does not take into account complications such as foreign body reactions (biomaterials, excess cement), or technical, mechanical, esthetic, or biological complications (peri-implant diseases).

## Conclusion

Implant survival was not significantly influenced by radiation therapy in this short observational period. In grafted bone implants, survival was significantly inferior than in native bone. Mucosal overgrowth around implants was more common in mucosal grafted areas versus nongrafted areas.
